# Influence of the Pressure Difference and Door Swing on Heavy Contaminants Migration between Rooms

**DOI:** 10.1371/journal.pone.0155159

**Published:** 2016-05-12

**Authors:** Jacek Hendiger, Marta Chludzińska, Piotr Ziętek

**Affiliations:** Warsaw University of Technology, Faculty of Building Services, Hydro and Environmental Engineering, Air Condition and Heating Department, Warsaw, Poland; Tsinghua University, CHINA

## Abstract

This paper presents the results of investigations whose aim was to describe the influence of the pressure difference level on the ability of contaminants migration between neighbouring rooms in dynamic conditions associated with door swing. The analysis was based on airflow visualization made with cold smoke, which simulated the heavy contaminants. The test room was pressurized to a specific level and then the door was opened to observe the trail of the smoke plume in the plane of the door. The door was opened in both directions: to the positively and negatively pressurized room. This study focuses on the visualization of smoke plume discharge and an uncertainty analysis is not applicable. Unlike other studies which focus on the analysis of pressure difference, the present study looks at the contaminants which are heavier than air and on “pumping out” the contaminants by means of door swing. Setting the proper level of pressure difference between the contaminated room and the neighbouring rooms can prove instrumental in ensuring protection against toxic contaminants migration. This study helped to establish the threshold of pressure difference necessary to reduce migration of heavy contaminants to neighbouring rooms.

## Introduction

An effective and well-designed ventilation system is essential when protection of indoor environment against external conditions is required, and on the premises of special use where hazardous airborne contaminants could be released. Control of contaminants migration between rooms or areas is crucial in both cases. The common strategy to direct the airflow and thus the contaminants transfer is room pressurization. It is widely utilized in chemical or biological laboratories and clean rooms. Room pressurization is also the main method used for the protection of isolation rooms in hospitals. In health care facilities, this way of ventilation system operation is utilized in both the Airborne Infection Isolation and Protective Environment Rooms, however, the key difference is the required direction of airflow between the room and the adjacent space, such as a corridor, which determines the application of negative or positive pressure in the protected room, respectively. Positive pressure ventilation is also designed in operating rooms.

There are many different suggestions with regard to the level of pressure difference which should be created to avoid contaminants migration between rooms. Useful information can be found in the guidelines for health services buildings, biological and chemical laboratories, as well as for smoke control systems, but the specific value depends on the local standards, law or recommendations.

### Steady-state conditions

According to the US Center for Disease Control and Prevention [1], a minimum pressure difference needed to direct airflow between rooms should be equal to 2.5 Pa. A similar value is recommended by the American Institute of Architects [2]. The UK Department of Health in the best practice guidance for health buildings recommends 5 Pa as a minimum for negative pressure isolation and, instead of a negative pressure in the isolation room, allows a positive pressure of outside corridor of 8–12 Pa (10 Pa nominally) [3].

Guidelines in Taiwan suggest a minimum negative pressure of 8 Pa in relation to the adjacent room or corridor [4]. ASHRAE [5] recommends 12.45 Pa as a value of pressure difference for the rooms of enhanced cleanliness requirements (class), which is confirmed by the study conducted by Ahmed et al. 1993 [6]. Similar guidelines can be found in the Guidance for Industry Sterile Drug Products [7], where, in the case of clean areas, the value of 10–15 Pa is recommended as a minimum value of pressure difference protecting the room. In the case of protection of aseptic isolations, where it is recommended to achieve complete physical separation from the external environment, the values range from 17.5 to 50 Pa.

Some publications refer to the airflow control as a primary means of protection of rooms and thus having an indirect influence on diversification of pressure between adjacent rooms NIH (2003) [8]; AIA (2001) [2]; Hitchings (1994) [9]; Gill (1994) [10] and Coogan [11]. Such publications recommend a range of 126–510 m^3^/h as the difference between the exhausted and supplied air in a contaminated room. However, since the value of the difference of airflow and the generated pressure difference value are closely connected, Streifel (2000) [12] recommends 212 m^3^/h as a difference in the airflow and simultaneously points out 2.5 Pa as a minimum pressure difference necessary to protect rooms and 7.5 Pa as an optimum value. Additionally, Gill [10] presents a minimum velocity on the room leakages at the level of 0.508 m/s. However, all the above guidelines and considerations refer to the steady-state conditions with the doors closed.

Another ratio recommended in the guidelines, especially for health care rooms, is the required air change per hour (ACH), which, depending on the source, ranges from 6 to 12 ACH [1, 3]. Research with tracer gas SF_6_ conducted by Tung et al. [4] indicates that the ventilation system of negative pressure differential 15.0 Pa in the isolation room demonstrates the best ventilation efficiency to extract contaminants, for both 12 and 24 ACH, which were tested. At lower values of pressure differential the more important factor was ACH, and higher ACH gives better results.

### Dynamic conditions

Despite meeting all the guidelines, it is difficult to entirely prevent incoming contamination. In practice, even with ventilation systems operating correctly, contaminants may migrate, which may have adverse human health effects. A very important factor, which can contribute to contamination migrating between the rooms despite pressure difference, is door operation [13, 14, 15, 16, 17, 18]. The problem is more important when frequency of door opening is high, which can be the case in operating rooms [19, 20]. Door opening immediately causes pressure equilibration between the rooms [21, 15, 22]. Moreover, it was demonstrated that door swinging makes the air mass from both sides blend, especially at the top edge of the door wing, while the difference in the airflow, which protects the rooms when the door is open, is too low to direct the airflow appropriately with the door open [15, 22]. Thus, it was noted that the door should not be opened rapidly. Similar conclusions can be found in [14] where it is pointed out that the negative pressure gradient may have been transiently reversed if the door-opening motion was too rapid, and sliding doors were recommended instead of hinged one.

Under the study [15, 22] permanent conditions with the door open were also verified. The limit value, which prevents the air from coming out of the room was the pressure difference at the level of 2 Pa. To compare, the American Industrial Hygiene Association [23] recommends minimum airflow velocity of 0.25 m/s through any opening, including open doorways, and preferred velocity of 0.51 m/s in a desired direction.

Airflow through the doorway is also influenced by the air density difference which results from temperature difference between neighbouring rooms [16, 17, 24, 25]. When the temperature difference is high enough, the airflow is directed by the gravity rather than by the pumping effect of door swing [16, 17].

The research described in the available literature, concerning migration of air between rooms in the conditions of pressure difference was conducted using tracer gases, CFD simulations and smoke visualization. It also took into consideration such factors as door operation, temperature difference or ACH level. However, most investigations focused on the general contaminant without taking into account its weight and location in the room.

Whenever it is required to protect rooms against migrating contamination, contamination may also contain elements which are heavier than air. In laboratories, these may be chemical compounds connected with the production taking place in the laboratory, while in hospitals these may be anesthetic gases or even aerosols containing hazardous bacteria or viruses. Research on heavy contaminants behaviour is also important due to the fact that among the heavier than air contaminants there are a lot of toxic substances belonging to the group of chemical weapons. [26].

Literature does not define specific requirements for the contaminants heavier than air, apart from the layout of air exhausts (if any). Therefore, in room protection focused design the same pressure difference values are usually used, regardless of the kind, density or weight of the contaminants.

In the present paper we focused on heavier-than-air contaminants, accumulated in the lower part of a room. The tests were made with dense smoke visualization in order to investigate the relation between the value of pressure difference, door swing and migration level of heavy contaminants between the rooms. In addition, disturbances in contaminant transfer resulting from movement of a person from a contaminated room to a protected one were also considered.

## Materials and Methods

The main method of conducting tests and observation was visualization of contaminated air flow, carried out by means of smoke.

### Experimental Facilities

**Fig 1:**
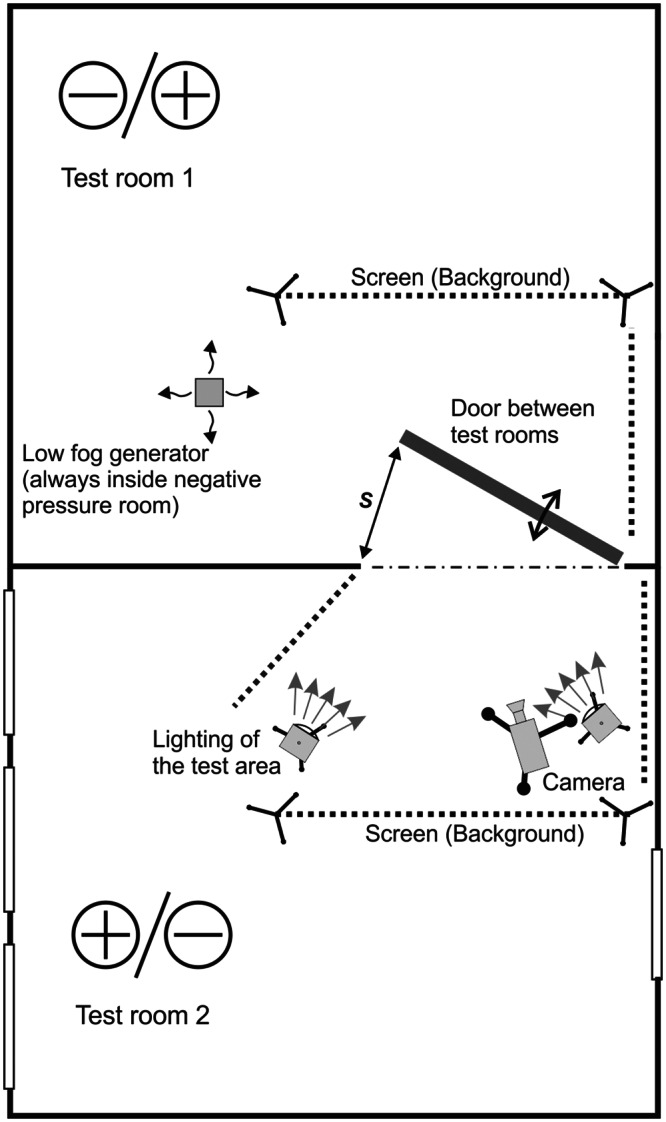
A scheme of the testing stand—testing rooms, top-view.

The tests were conducted on a suitably prepared testing stand. The measuring stand was configured in a testing room equipped with air-supply/air-exhaust systems (Fig 1). Both adjacent rooms, where the tests were conducted, had an area of 6x8 m and were 3 m high. They were connected by a single-wing swing door, 2x0.9 m in size.

The measuring set-up let us precisely set and monitor the pressure difference between test rooms, measure velocity of the air in the apertures, airflow rate of the air supplied or exhausted from the rooms and visualize the contaminant transfer. A set of measurement series was performed with different values of maintained pressure difference, starting with small values, i.e. 2.5 Pa till 50 Pa used in standard smoke control systems.

**Fig 2:**
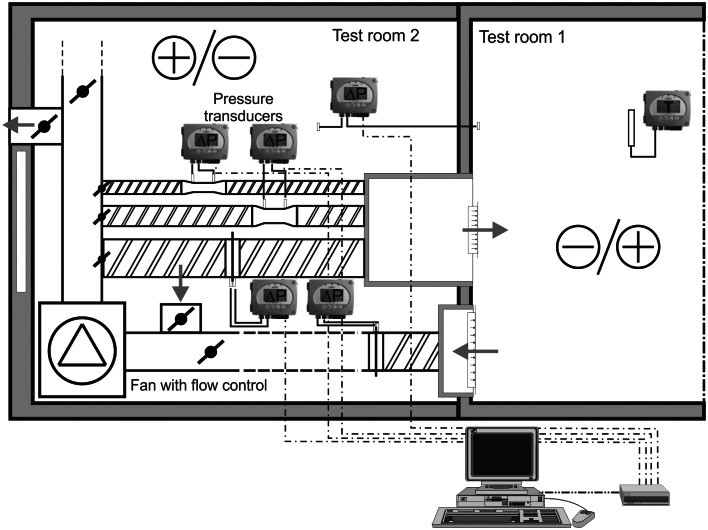
A scheme of measuring stand—ventilation system with measurement and adjustment elements.

The main components of the test installation were (Fig 2):

- a device for adjustment and measurement of supply/exhaust airflow rate, adjustment dampers, measuring area reducers and orifice plate, pressure transducers,
- simulated source of smoke—the low fog machine,
- camera for visualization of smoke transfer with adjustment elements,
- lighting for measurement area.

The area near the door in both test rooms was lined with black fabric in order to enhance contrast between the smoke and the background. The smoke, generated by the low fog machine, was let in, each time, just above the floor level, at the distance of approx. 1.5 m from the doors.

The airflow volume was measured by a system containing a measuring tube (Venturi flow meter) and a pressure transducer. The measuring tube was a standard made element with external diameter of 160mm and measurement accuracy not lower than 5%. The pressure transducer connected with the measuring tube had a measuring range from 0 to 500 Pa, resolution of 1 Pa and measurement accuracy of 0.5%.

The airflow volume was calculated as follows:

$$\text{V}\;=\;\text{k}\cdot \sqrt{\Delta P_{m}}$$ (1)

where:

k—constant value for the measuring tube,

ΔPm—measurement pressure, Pa.

The maximum measurement error of the airflow volume V, calculated as an error of a compound value was 5.3%.

Positive pressure in the room was measured by means of a pressure transducer with measuring range of 0–100 Pa, resolution of 0.1 Pa and measurement accuracy of 0.5%.

During all measurement sessions a constant temperature of 23°C was maintained both in the rooms and in the ventilation system which generated pressure difference.

### Air leakage characteristics of the test room

Pressure difference between the rooms provoked air leakage through the air leakage points in the room envelope, and in the closed elements of the ventilation system. Functional relationship between the airflow volume and the pressure difference it generated as well as geometry of the openings, the so called Power law equation, confirmed in numerous sources in the literature, was proposed by ASHRAE in [27]:

$$Q\;=\;c\;\cdot \;{\left(\Delta p\right)}^{n},$$ (2)

where:

Q—airflow through opening, m^3^/s,

c—flow coefficient, m^3^/(s ·Pa^n^),

n—pressure exponent, [–],

Δp—pressure difference across opening, Pa.

Exponent (n) and coefficient (c) depend on the kind of openings through which the air passes. The research conducted [28] also proved that the results obtained depend significantly on the geometrical parameters of the openings and on the inflow and outflow of air from the opening. The value of exponent (n) on the basis of tests performed by Walker [29] for the process of air infiltration through the openings, is constant and equals approx.0.6–0.7. Most frequently, exponent (n) has a typical value of 0.65. The value of flow coefficient (c) and that of pressure exponent (n) can be determined empirically by means of appropriate pressure tests.

During the tests parameters of the test room were determined, with the door and other openings closed. For this purpose, volume of the airflow supplied to the test room with gradually increasing pressure values was measured. The results of airflow measurements are shown in Table 1.

**Table 1:** Results of the airflow volume measurements and of the positive pressure generated during the tests of the room leaktightness.

Positive pressure in test room [Pa]	Airflow volume [m^3^/h]	Airflow volume [m^3^/s]
0	0	0
2.5	390	0.108
5	725	0.201
7	880	0.244
10.5	1035	0.288
12.5	1128	0.313
16	1336	0.371
25	1933	0.537
50	2830	0.786

The above results were approximated by means of the function described in Eq 1.

The following relationship was obtained:

$$Q=0,067\cdot \Delta P^{0,63}$$ (3)

with correlation coefficient R = 0.998

Adopting a typical coefficient n = 0.65 resulted in a very good representation of room characteristics in the form of the following relationship:

$$Q=0,063\cdot \Delta P^{0,65}$$ (4)

with correlation coefficient R = 0.998

**Fig 3:**
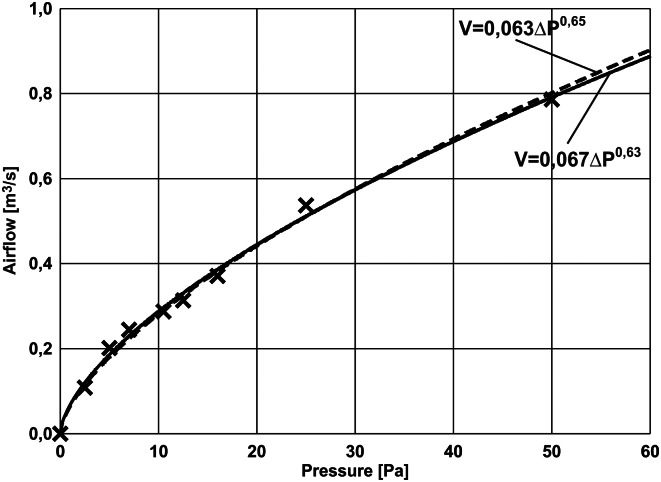
Airflow rate versus pressure difference data from pressurization test.

Room characteristics are shown in Fig 3.

As it can be seen, the test room used in the test has typical characteristics describing leakage.

### Test range and procedure

The tests were performed in measurement series with different values of maintained initial level of pressure difference (from low values of approx. 2.5 Pa to the values typically used in desmoking systems, i.e. 50 Pa) and the direction and width of the door swing shown in Tab.2.

The testing variants assumed certain behavioural patterns of the users. For this reason, the case of opening and closing the door adjacent to the contaminated area was considered, i.e. a scenario in which a person is looking out of the door in order to assess the situation.

For this testing variant, two widths of door swing were taken into account i.e. 10 cm (s = 10) and 50 cm (s = 50). Variant s = 10 was realized at two values of door swing velocity, the so called „slow” and „fast”. Moreover, during the measurement series the influence exerted by movement of a person on the contaminants migration was also taken into consideration. Such a scenario corresponds to the case of “escape” from the endangered zone. However, with variants s = 10 and s = 50 the door was opened and then closed while in the escape mode the door was opened to 50 cm and left open after a person transition. The process of opening and closing the door was manual so there could have occurred small differences in velocity and time of its opening. Testing variants used in measurement series are shown in Table 2.

**Table 2:** Summary of the test series.

Case	I (Door swing towards negatively pressurized zone)				II (Door swing towards positively pressurized zone)			
Variant	s = 10 fast	s = 10 slow	s = 50	„escape” mode	s = 10 fast	s = 10 slow	s = 50	„escape” mode
Width of door swing [cm]	10^a^	10^a^	50^b^	50^b^	10^a^	10^a^	50^b^	50^b^
Time of the door opening[sec.]	1.5	3	3.5	1.5 (only opening of the door)	1.5	3	3.5	1.5 (only opening of the door)
Value of the generated pressure difference between rooms [Pa]	2.5, 5.0, 7.0, 12.5, 25.0, 50.0							

^a^ The width of the door swing corresponds to the opening angle of approx. 6–8°.

^b^ The width of the door swing corresponds to an angle of approx. 32–36°.

Simulation of contamination was carried out using a low fog machine. During measurements the machine was always placed on the negatively pressurized side, approx. 1.5 m away from the door.

Smoke was introduced at the floor level (5 cm above the floor) in a low turbulent manner, so the mixing of smoke with indoor air was limited. The height of smoke layer was about 10–15 cm at the beginning of each test. Temperature of air above the smoke layer in the negative pressure room was equal to the air temperature in the positive pressure room, and no significant temperature gradient was observed in both rooms.

The course of a single measurement series was:

- closing of the door,
- generating appropriate pressure difference,
- start-up of a simulated source of contamination (low fog machine),
- generating appropriate conditions: opening and closing of the door, “escape” mode.

## Results

Selected results obtained in different measurement series and notes about each series are presented below. Case „I” refers to the door opening towards the negatively pressurized zone, while case”II” refers to the door opening towards the positively pressurized zone. Descriptions of the series and conclusions were prepared on the basis of visualization and observation of the smoke movement. Temperature during the measurements remained constant, therefore, the influence of the buoyant force on contamination movement through the door can be excluded. The door was opened manually and therefore small differences in velocity and time of door opening may have occurred. However, control measurements showed that these differences would not have been greater than 5%.

**Fig 4:**
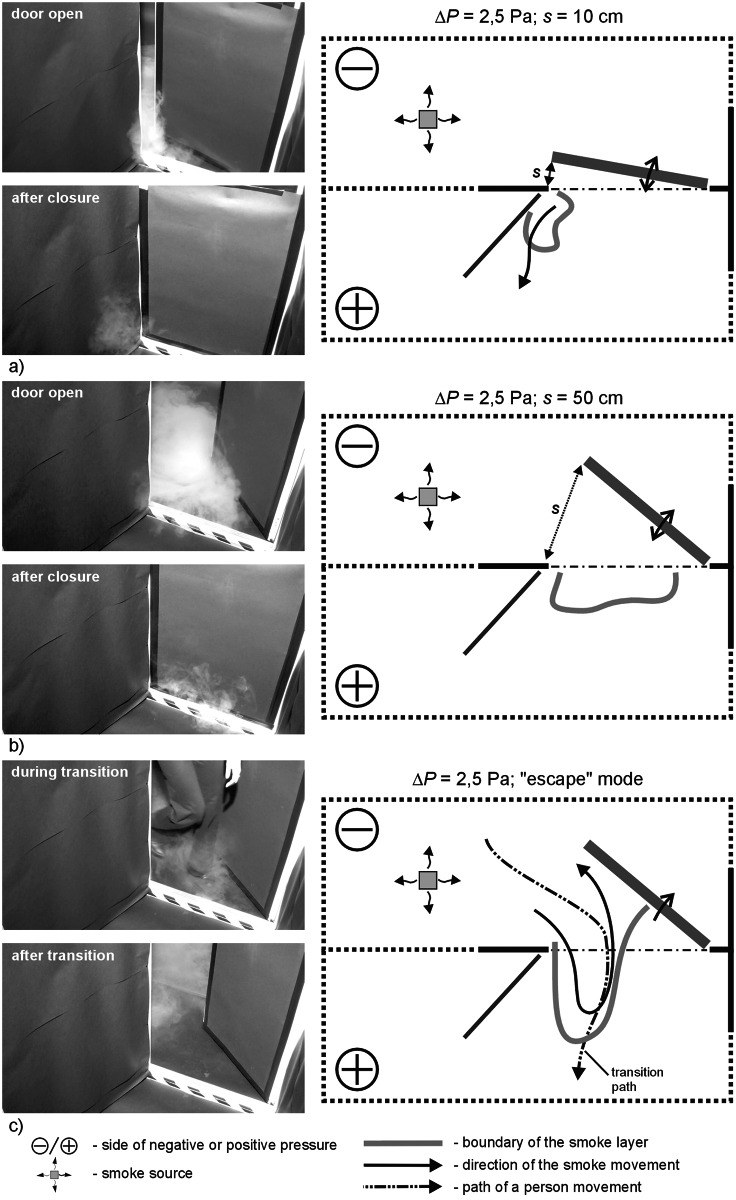
Results of visualization performed with the door swing towards negatively pressurized zone—case I, Δp = 2,5 Pa, a) 10cm opening width, b) 50cm opening width, c) „escape” mode.

**Fig 5:**
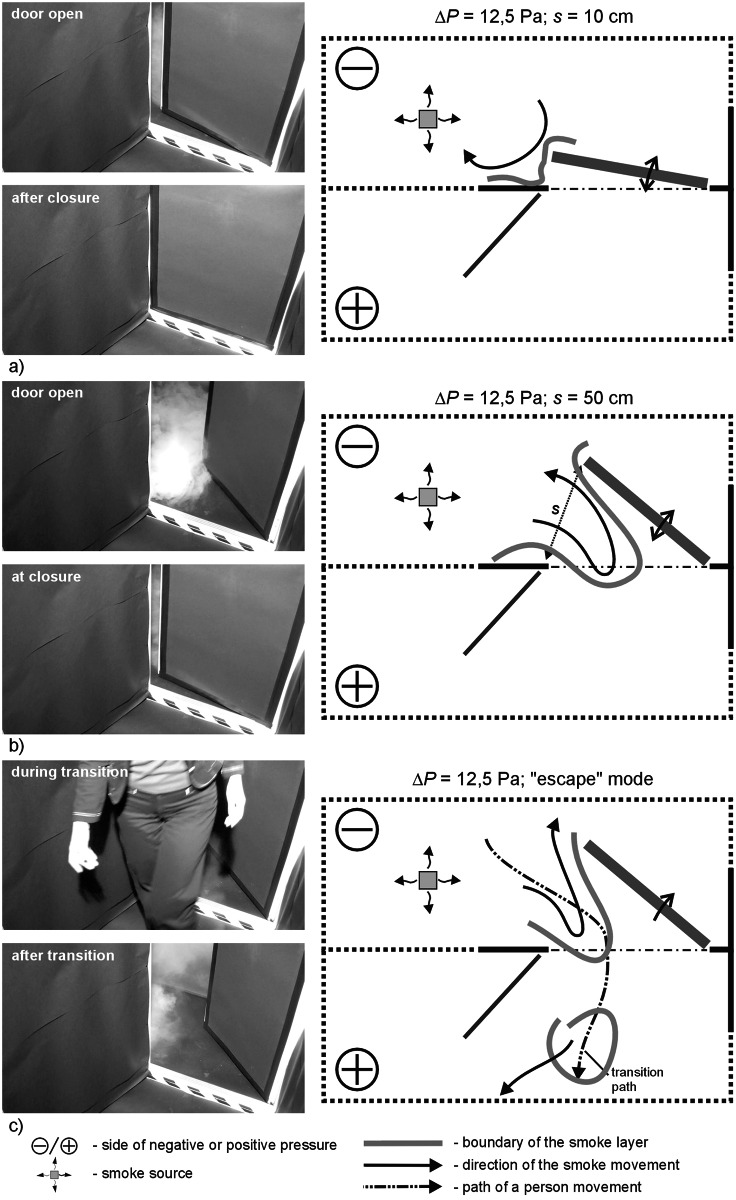
Results of visualization performed with the door swing towards negatively pressurized zone—case I, Δp = 12.5Pa a) 10cm opening width, b) 50cm opening width, c) „escape” mode.

**Fig 6:**
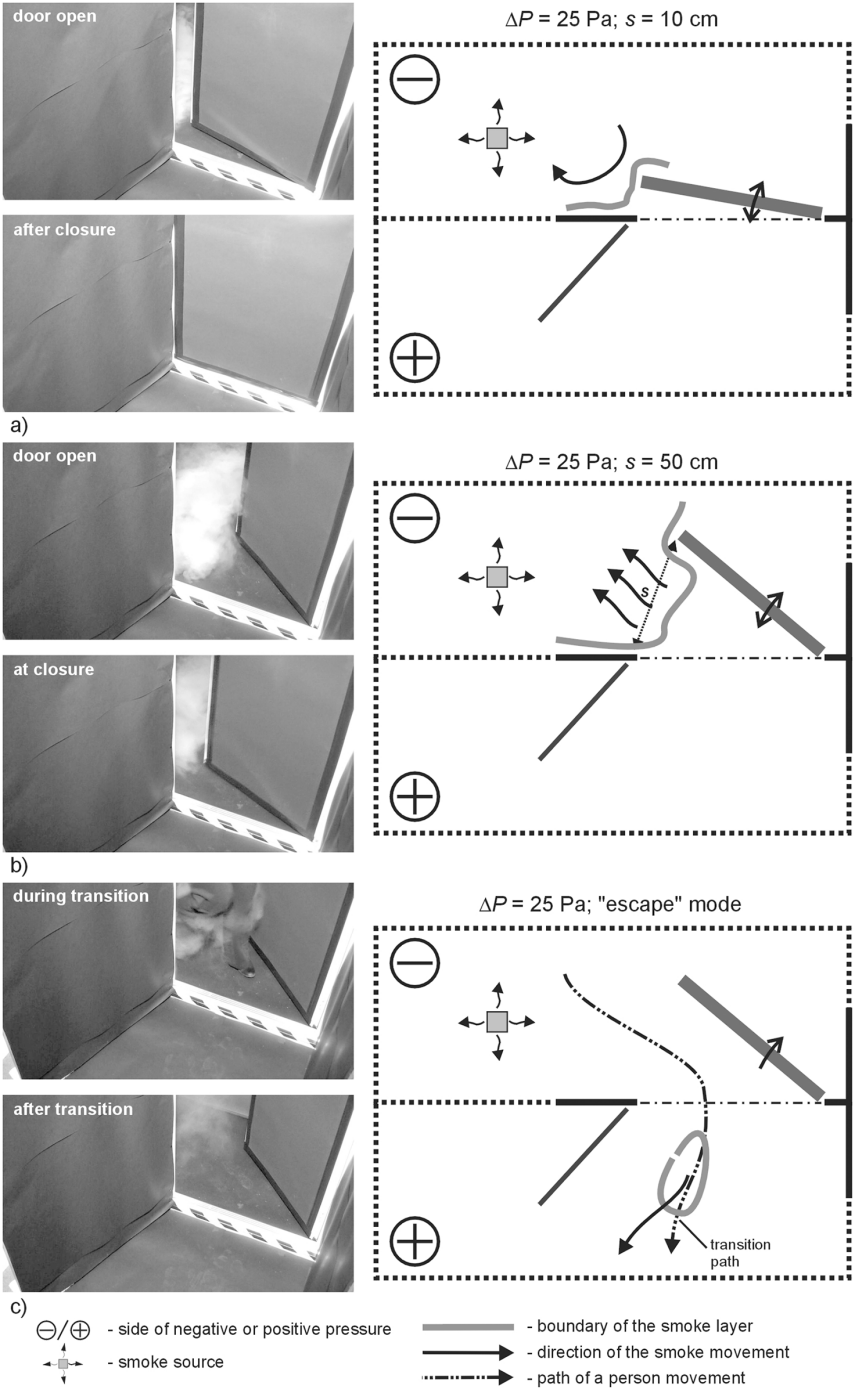
Results of visualization performed with the door swing towards negatively pressurized zone- case I, Δp = 25 Pa a) 10cm opening width, b) 50cm opening width, c) „escape” mode.

**Fig 7:**
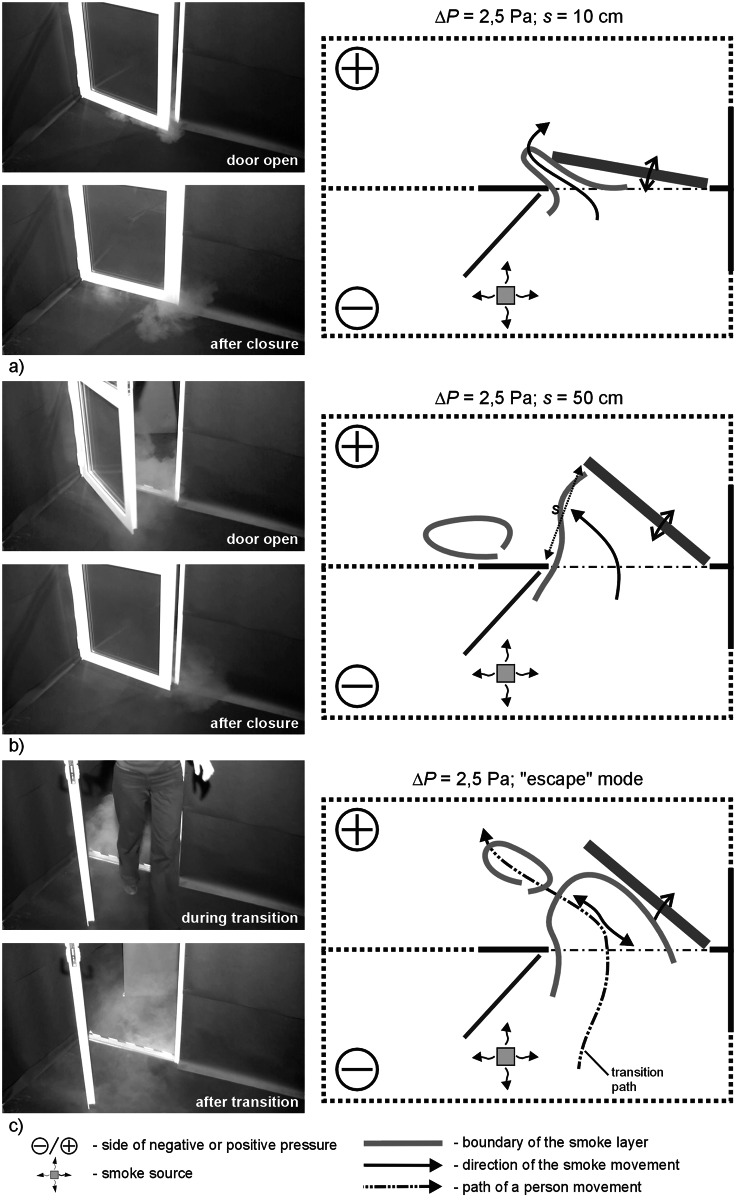
Results of visualization performed with the door swing towards positively pressurized zone—case II, Δp = 2.5 Pa a) 10cm opening width, b) 50 cm opening width, c) „escape” mode.

**Fig 8:**
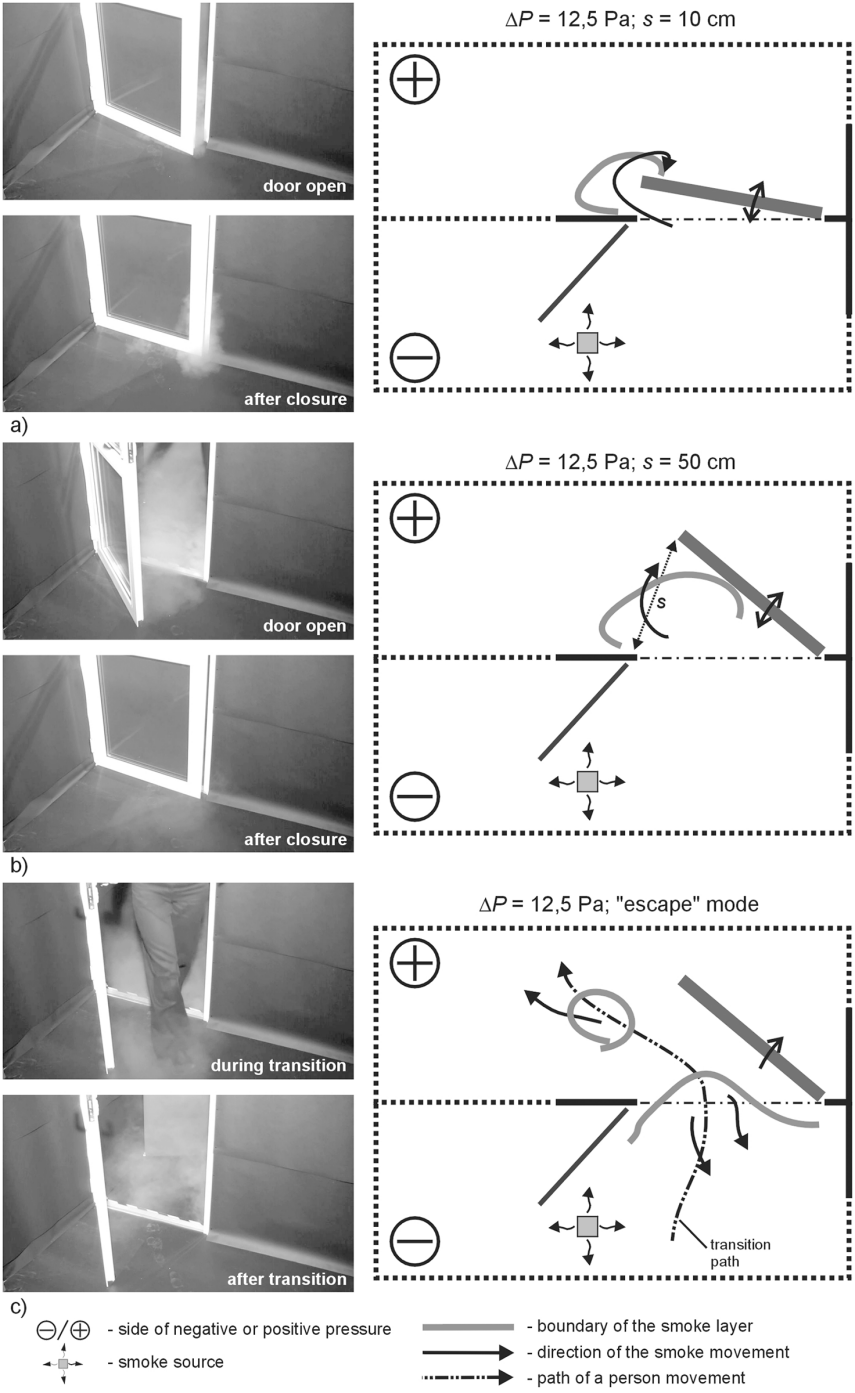
Results of visualization performed with the door swing towards positively pressurized zone—case II, Δp = 12.5 Pa a) 10cm opening width, b) 50 cm opening width, c) „escape” mode.

**Fig 9:**
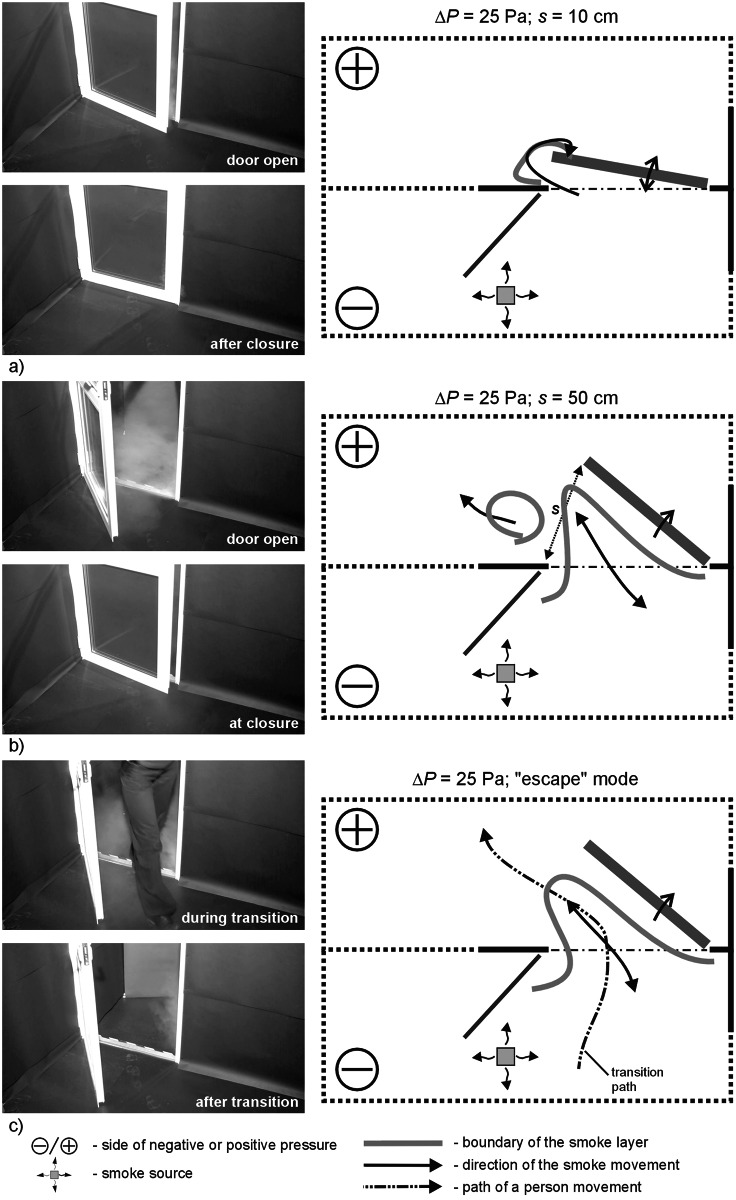
Results of visualization performed with the door swing towards positively pressurized zone—case II, Δp = 25 Pa a) 10cm opening width, b) 50cm opening width, c) „escape” mode.

Figs 4–9 shows the results for cases I and II and pressure difference: 2.5, 12.5 and 25 Pa.

### Results of visualization performed with the door swing towards negatively pressurized zone

In all the cases, with 2.5 Pa pressure difference, a considerable amount of smoke migrated to the adjacent room with the door closed. In the “escape” mode, a plume of smoke trailed after the person leaving the room. However, when the person passed through the door and left it open, the smoke nearby the door tended to withdraw.

With 12.5 Pa, when the door was opened and closed at the width of 10 cm, the smoke practically did not migrate out of the room. At 50 cm wide opening and in the “escape” mode, a relatively small amount of smoke remained outside the room.

Above the pressure difference of 25 Pa, the smoke practically did not migrate out of the room when the door was opened and closed. Only at 25 Pa, a passage of a person still caused moderate migration of the smoke outside.

### Results of visualization performed with the door swing towards positively pressurized zone

In all the cases, with pressure difference up to 5 Pa, the smoke remained outside the room after the door was closed. In the “escape” mode the smoke trailed after the person leaving the room.

A 25 Pa the degree of smoke migration when the door was closed and opened, fell to a moderate level. When a person passed through the door, the smoke practically did not trailed after the person and soon withdrew.

### Comparison of case I and case II results

**Fig 10:**
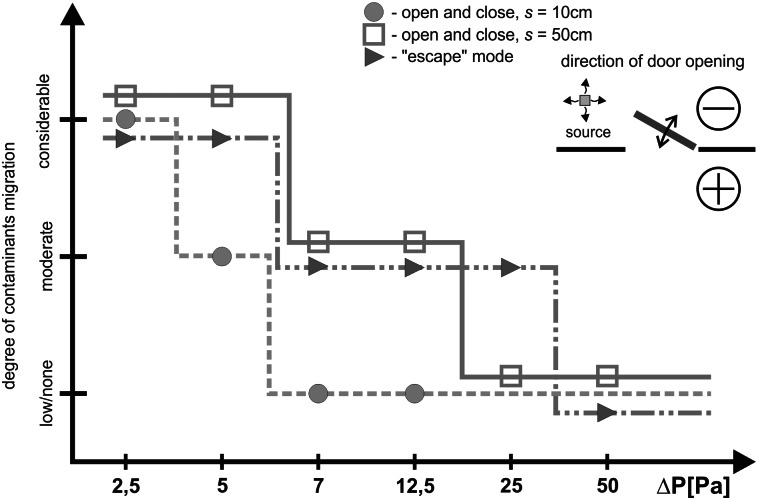
Level of contaminants migration—door open towards negatively pressurized zone.

**Fig 11:**
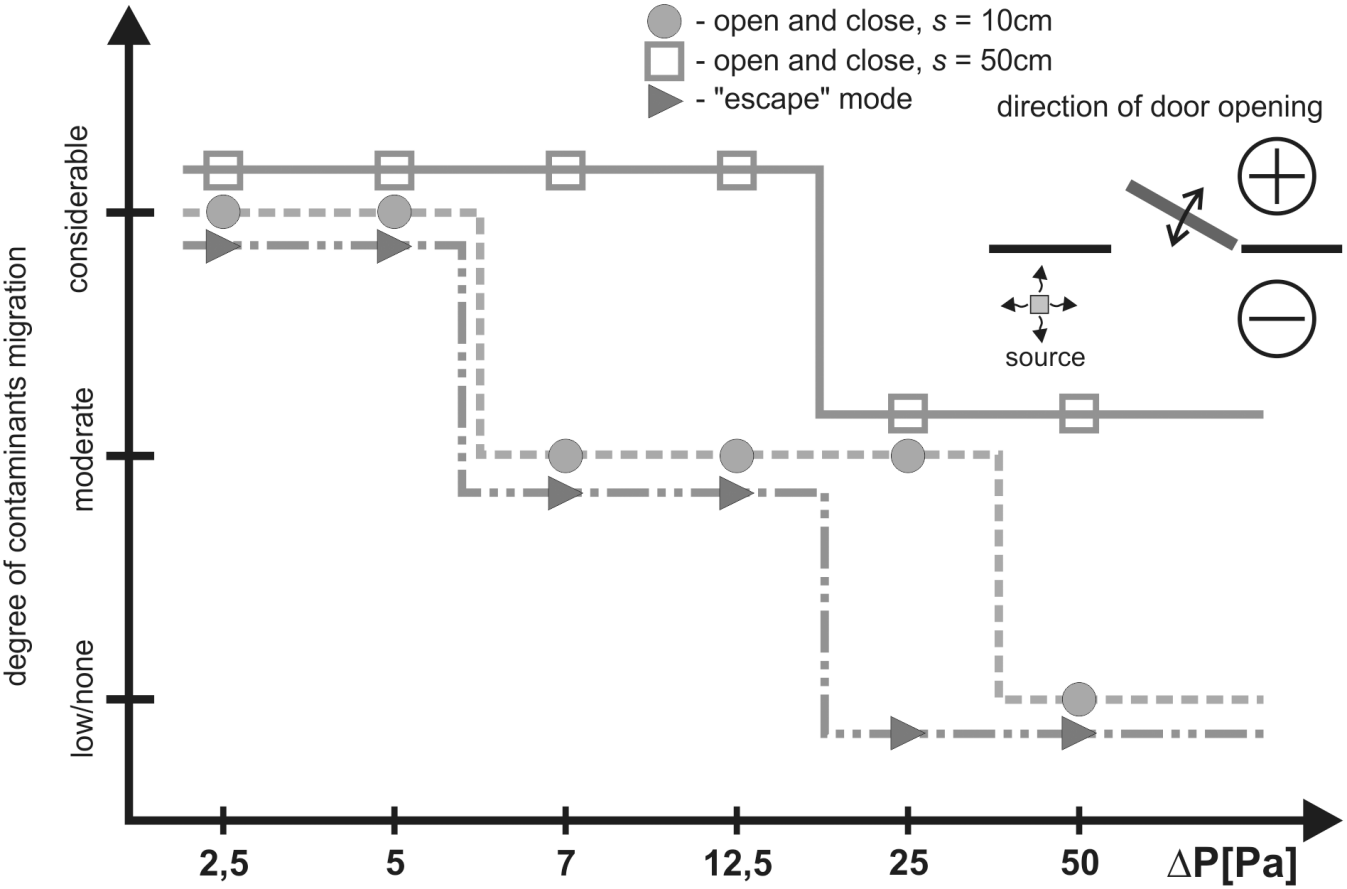
Degree of contaminants migration—door open towards positive pressure.

Figs 10 and 11 show the results obtained in different series. During the measurement series with the door opened towards a negatively pressurized zone (Fig 10), in the case of the lowest values of pressure difference (2.5 Pa), a considerable degree of contaminants migration to the room was observed. After closing the door, it was noted that a certain portion of the smoke left migrated further towards an adjacent room or remained at the doorsill. Increased pressure difference (5, 7, 12.5 Pa) between rooms made it possible to arrest smoke in a room when the door was open to the width of 10 and 50 cm. However, a passage of a person through the door resulted in intensive turbulences causing discharge of certain portion of smoke plume outside.

When the door was left ajar, the airflow provoked by generating initial pressure difference of 25 Pa, prohibited smoke from getting outside. The same case was noted in previous measurement scenarios. Additionally, like in other cases, also in those conditions, the opening of the door and passage of a person through that door resulted in a small discharge of smoke plume. Initial pressure difference generated at the level of 50 Pa caused a complete blockage of smoke transfer even when a person was passing through the door, not only when the door was partly open.

During the measurements conducted with the door directed towards a positive pressure zone (Fig 11) a slightly bigger smoke plume discharge was noted in comparison to the corresponding measurements for the door swing towards negative pressure. Pressurization at the level of 2.5 Pa, 5.0 Pa, irrespective of the width of the door swing, does not prevent migration of the smoke plume outside, which remains in a protected room after the door has been closed. It is only with the door left ajar that a slow withdrawal of smoke plum to a contaminated room was observed. A similar phenomenon was noted during the measurement series with pressure difference of 7.0 and 12.5 Pa; the amount of smoke plume migrating outside was visibly smaller.

In comparison to the measurements with lower pressurization, initial generation of pressure difference at 25 Pa noticeably minimizes migration of smoke plume outside, irrespective of the width of the door swing and of a passage of a person through the door. Only minute quantities of smoke migrate.

## Discussion

Opening of the door between the rooms of different pressure levelled pressure value on both sides, which was proved by the tests performed, and confirmed in some publications [21, 15, 22].

The studies showed that door swing outside the room towards a positively pressurized zone was more disadvantageous in comparison to the cases when the door was opened into the contaminated room (with negative pressure). With the door opened towards negative pressure, reduction of the amount of contamination to the level specified as moderate or small occurred when the pressure difference was at approx. 7 Pa. That was the case for all the examined modes of door swing (s = 10 fast, s = 10 slow, s = 50, “escape” mode). Similar reduction in the amount of contamination in the case of opening of the door towards positive pressure was observed only with the door swing at 12.5 Pa, and even at 25 Pa. This finding is confirmed by the studies of Sansone and Keimig [15] published earlier.

The reason for such a phenomenon is the effect of drawing in contamination through the opposite to the leading side of the door opening towards the room with positive pressure and further pushing of the contamination into the room when the door was closed. In the case of door swing towards the negative pressure, the smoke was drawn in through the eddies that formed around the edge of travelling door, but the air passing through the door left ajar made the smoke withdraw. Different observations were recorded by Wiesman in [22], where the author decided that it is more advantageous to open the door towards the positive pressure. However, his studies assumed only opening of the door in the required direction without closing the door afterwards, which did not take into account the door pumping effect.

This effect has a considerable impact on contamination spreading. The studies performed showed that the amount of contamination depends on the velocity of contamination pumping. Faster pumping made more contamination migrate. This was demonstrated by both measurement sessions for cases II 12.5 and 50 Pa carried out in variants s = 10 slow and s = 10 fast, and also by the comparison of the „escape” mode and s = 50 mode. In both cases longer time of door swing was connected with a smaller amount of contamination getting out. These observations confirm the conclusions put forward by Sansone and Keimig [15] showing that it is necessary to swing the door more slowly.

Door pumping presents a considerable difficulty for the effective protection against contamination migration. The tests show that it causes greater contaminants migration as compared to the door left ajar in stable conditions. The measurements performed showed that in order to protect the rooms against contaminants migration during door pumping, it is necessary to have an airflow ensuring velocity of approx. 0.6–1.0 m/s on the door left ajar. It is a three times greater value in comparison with the values recommended by the AIHA [2] in the case of the door left ajar in stable conditions (0.254m/s).

Moreover, measurement results presented in the article indicate that contaminants migration depends on two factors: door pumping and transferring contaminants on the feet of the person passing through the door in the „escape” mode. However, with a lower pressure difference and with lower velocity values with the door left ajar, door pumping plays a much bigger role. With the pressure and velocity values growing with the door left ajar, the influence of door pumping decreases, while the contamination is transferred mainly on the person’s feet.

## Conclusions

The presented studies, unlike the other ones which also examine the influence of pressure difference, included the “pumping” effect, i.e. transfer of contaminants by means of the door swing. The observations provided the grounds to conclude that the door effect can be a contributing factor to intensification of contaminants transfer, even in comparison to the cases when the door was left open.

Although, in terms of contamination, it is more advantageous to swing the door towards negative pressure of 2.5 Pa, as shown in some publications, it is definitely not sufficient in order to protect the rooms if the door swings. Door swinging towards negative pressure causes reduction in the contaminants transfer to the level specified as moderate or small already at the pressure of 7 Pa. The same effect with the door opened in the opposite direction is obtained with pressure difference of 12.5 Pa and even 25 Pa. Increase of pressure difference from 25 to 50 Pa did not help to prevent migration with the door swing towards negative pressure.

Moreover, it was shown that quick door swing causes a greater transfer of contaminants regardless of the width of the door opening, which shows that it is necessary to open the door more slowly.

In order to protect the rooms against contaminants transfer during door pumping it is necessary to have the airflow which guarantees velocity of approx. 0.6–1.0 m/s on the door left ajar.

Contaminants migration is also influenced by their transfer on the feet of the person passing through the door. With an increase in pressure and velocity on the door left ajar, the influence of door swing decreases while the effect on contamination transfer on the feet increases.

## References

[1] Centers for Disease Control and Prevention (US). MMWR (Morbidity and Mortality Weekly Report): Guidelines for preventing transmission of mycobacterium tuberculosis in health-care settings. 2005; 54:NRR-17. 16382216

[2] American Institute of Architects. Guidelines for the design and construction of hospital and health care facilities, 2001.

[3] UK Department of Health. Health Building Note (HBN) 04–01 Supplement 1: Isolation facilities for infectious patients in acute settings. 2005.

[4] Tung YC, Hu SC, Tsai TI, Chang IL. An experimental study on ventilation efficiency of isolation room. Building and Environment 2009; 44(2): 271–279. 10.1016/j.buildenv.2008.03.003

[5] American Society of Heating, Refrigerating, and Air-Conditioning Engineers Inc. ASHRAE Handbook—HVAC Applications. Atlanta, 2007.

[6] Ahmed O, Mitchell JW, Klein SA. Dynamics of laboratory pressurization, ASHRAE Transactions, 1993; 99(2): 223–229.

[7] US Food and Drug Administration, FDA Center for Drug Evaluation and Research, Center for Biologics Evaluation and Research and Office of Regulatory Affairs. Guidance for Industry, Sterile Drug Products Produced by Aseptic Processing—Current Good Manufacturing Practice. Washington, DC 2004.

[8] US Department of Health and Human Services, National Institute of Health (US), Office of Research Facilities. NIH Design Policy and Guidelines. 2003.

[9] Hitchings DT. Laboratory space pressurization control systems. ASHRAE Journal. 1994; 36(2): 36–40.

[10] Gill KE. HVAC design for isolation rooms. Heating, Piping and Air Conditioning Engineering. 1994; 66(2): 45–52.

[11] Coogan JJ. Effects of surrounding spaces on rooms pressurized by differential flow control. ASHRAE Transactions. 1996; 102(1): 18–25.

[12] Streifel AJ. Health-care IAQ: guidance for infection control. Heating, Piping, Air Conditioning Engineering. 2000; 72(10): 28–36.

[13] Gustavsson N. Dispersion of small particles into operating rooms due to door openings—A measurement study performed at Sahlgrenska. M.Sc. Thesis, University Hospital in Göteborg Department of Energy and Environment, Division of Building Services Engineering, Chalmers University Of Technology, Göteborg, Sweden. 2010.

[14] Tanga JW, Eamesb I, Lic Y, Tahad YA, Wilsone P, Bellinganf G, et al Door-opening motion can potentially lead to a transient breakdown in negative-pressure isolation conditions: the importance of vorticity and buoyancy airflows. Journal of Hospital Infection. 2005; 61: 283–286. 16253388 10.1016/j.jhin.2005.05.017 PMC7114940

[15] Sansone EB, Keimig SD. The influence of door swing and door velocity on the effectiveness of directional airflow. Proceedings of ASHRAE IAQ '87. 1987: 372–381.

[16] Kiel DE, Wilson DJ. Combining Door Swing Pumping with Density Driven Flow. ASHRAE Transactions. 1989; 95(2): 590–599.

[17] Wilson DJ, Kiel DE. Gravity Driven Counterflow Through an Open Door in a Sealed Room. Building and Environment. 1990; 25(4): 379–388.

[18] Tang JW, Nicolle A, Pantelic J, Klettner CA, Su R, Kalliomaki P, et al Different types of door-opening motions as contributing factors to containment failures in hospital isolation rooms. PloS one. 2013 6 24;8(6):e66663 10.1371/journal.pone.0066663 23826109 PMC3691190

[19] Smith EB, Raphael IJ, Maltenfort MG, Honsawek S, Dolan K, Younkins EA. The Effect of Laminar Air Flow and Door Openings on Operating Room Contamination. The Journal of arthroplasty. 2013; 28(9): 1482–1485. 10.1016/j.arth.2013.06.012 23890828

[20] Ljungqvist B, Reinmüller B, Gustén J, Gustén L, Nordenadler J. Contamination risks due to door openings in operating rooms. European Journal of Parenteral & Pharmaceutical Sciences. 2009; 14(4): 97–101.

[21] Shiha YC, Chiu CC, Wang O. Dynamic airflow simulation within an isolation room. Building and Environment. 2007; 42: 3194–3209. 10.1016/j.buildenv.2006.08.008 PMC7116969 32287999

[22] Wiseman B. Room pressure for critical environment. ASHRAE Journal. 2003;45(2): 34–39.

[23] ANSI/AIHA Z9.5–2003. American National Standard for Laboratory Ventilation. American National Standards Institute, Inc. American Industrial Hygiene Association. 2003.

[24] Chen C, Zhao B, Yang X. Impact of two-way air flow due to temperature difference on preventing the entry of outdoor particles using indoor positive pressure control method. Journal of hazardous materials. 2011 2 28;186(2):1290–1299. 21185117 10.1016/j.jhazmat.2010.12.003

[25] Chen C, Zhao B, Yang X, Li Y. Role of two-way airflow owing to temperature difference in severe acute respiratory syndrome transmission: revisiting the largest nosocomial severe acute respiratory syndrome outbreak in Hong Kong. Journal of the Royal Society Interface. 2010 11 10:rsif20100486. 10.1098/rsif.2010.0486 PMC3061095 21068029

[26] Mizielinski B, Hendiger J, Chludzinska M. Chemically polluted ventilating air as security hazard in terrorist attack. District Heating, Heating, Ventilation. 2010; 2: 35–36. Polish

[27] American Society of Heating, Refrigerating, and Air-Conditioning Engineers Inc. ASHRAE Handbook—Fundamentals. Atlanta, 2009.

[28] Chastain JP, Colliver DG, Winner PW. Computation of discharge coefficients for laminar flow in rectangular and circular openings. ASHRAE Transactions. 1987; 93(2): 2259–2283.

[29] Walker IS, Wilson DJ, Sherman MH. A comparison of the power law to quadratic formulations for air infiltration calculations. Energy and Buildings. 1997; 27(3): 293–299.

